# The Sequence of Chemotherapy and Toripalimab Might Influence the Efficacy of Neoadjuvant Chemoimmunotherapy in Locally Advanced Esophageal Squamous Cell Cancer—A Phase II Study

**DOI:** 10.3389/fimmu.2021.772450

**Published:** 2021-12-06

**Authors:** Wenqun Xing, Lingdi Zhao, Yan Zheng, Baoxing Liu, Xianben Liu, Tiepeng Li, Yong Zhang, Baozhen Ma, Yonghao Yang, Yiman Shang, Xiaomin Fu, Guanghui Liang, Dongfeng Yuan, Jinrong Qu, Xiaofei Chai, He Zhang, Zibing Wang, Hongwei Lin, Liang Liu, Xiubao Ren, Jiangong Zhang, Quanli Gao

**Affiliations:** ^1^ Department of Thoracic Surgery, Cancer Hospital Affiliated to Zhengzhou University and Henan Cancer Hospital, Zhengzhou, China; ^2^ Department of Immunotherapy, Cancer Hospital Affiliated to Zhengzhou University and Henan Cancer Hospital, Zhengzhou, China; ^3^ Department of Radiology, Affiliated Cancer Hospital of Zhengzhou University and Henan Cancer Hospital, Zhengzhou, China; ^4^ Department of Pathology, Affiliated Cancer Hospital of Zhengzhou University and Henan Cancer Hospital, Zhengzhou, China; ^5^ Department of Biotherapy, Tianjin Medical University Cancer Institute and Hospital, Tianjin, China; ^6^ Department of Cancer Epidemiology, Affiliated Cancer Hospital of Zhengzhou University and Henan Cancer Hospital, Zhengzhou, China

**Keywords:** toripalimab, chemoimmunotherapy, sequence, esophageal squamous cell carcinoma, pathological complete response

## Abstract

**Background:**

There is no standard neoadjuvant therapy for locally advanced esophageal cancer in China. The role of neoadjuvant chemotherapy plus immunotherapy for locally advanced esophageal cancer is still being explored.

**Methods:**

This open-label, randomized phase II study was conducted at a single center between July 2019 and September 2020; 30 patients with locally advanced esophageal squamous cell carcinoma (ESCC) (T3, T4, or lymph-node positive) were enrolled. Patients were randomized according to the enrollment order at a 1:1 ratio to receive chemotherapy on day 1 and toripalimab on day 3 (experimental group) or chemotherapy and toripalimab on day 1 (control group). The chemotherapeutic regimen was paclitaxel and cisplatin. Surgery was performed 4 to 6 weeks after the second cycle of chemoimmunotherapy. The primary endpoint was pathological complete response (pCR) rate, and the secondary endpoint was safety and disease-free survival.

**Results:**

Thirty patients completed at least one cycle of chemoimmunotherapy; 11 in the experimental group and 13 in the control group received surgery. R0 resection was performed in all these 24 patients. Four patients (36%) in the experimental group and one (7%) in the control group achieved pCR. The experimental group showed a statistically non-significant higher pCR rate (*p* = 0.079). PD-L1 combined positive score (CPS) examination was performed in 14 patients; one in the control group had a PD-L1 CPS of 10, and pCR was achieved; the remaining 13 all had ≤1, and 11 of the 13 patients received surgery in which two (in the experimental group) achieved pCR. Two patients endured ≥grade 3 adverse events, and one suffered from grade 3 immune-related enteritis after one cycle of chemoimmunotherapy and dropped off the study. Another patient died from severe pulmonary infection and troponin elevation after surgery.

**Conclusions:**

Although the primary endpoint was not met, the initial results of this study showed that delaying toripalimab to day 3 in chemoimmunotherapy might achieve a higher pCR rate than that on the same day, and further large-sample clinical trials are needed to verify this.

**Clinical Trial Registration:**

ClinicalTrials.gov, identifier NCT 03985670.

## Background

Esophageal squamous cell carcinoma (ESCC) is one of the common malignancies that threaten the health and life of human beings, especially for the Chinese (1). The standard treatment for patients at the early stage is radical surgery. Patients with locally advanced ESCC have a high rate of recurrence. At present, the standard treatment for locally advanced ESCC is simultaneous chemoradiotherapy (2, 3). With the race differences between the East and West, neoadjuvant chemotherapy is usually applied in Japan and China (4, 5). Immune checkpoint inhibitor represented by anti-PD-1 antibody has become one of the important regimens in advanced malignancies. Anti-PD-1 antibody plus chemotherapy could prolong the survival time of patients with advanced ESCC and has become the standard treatment for advanced ESCC (6).

Previous studies have shown a synergistic efficacy of chemotherapy and anti-PD-1 antibodies. Chemotherapy could induce immunogenic tumor cell death, deplete myeloid immunosuppressive cells selectively and lymphopenia, which reduces regulatory T (Treg) cells, and make room for proliferation of effect T cells (7). Thus, the combined application of these two agents has synergistic effects. Presently, chemoimmunotherapy combinations have comprised merely standard-of-care chemotherapy regimens being added to immunotherapy and then being compared with chemotherapy plus placebo, but the elements in this combination have not been optimized. For example, a randomized phase III study concluded that the addition of chemotherapy to pembrolizumab worsened survival over pembrolizumab or chemotherapy as a single treatment in patients with PD-L1 combined positive score (CPS) > 10 (8). Simultaneous administration of anti-PD-1 antibody and anti-OX40 antibody could reduce the antitumor effect of the anti-OX40 antibody; in contrast, administration of anti-PD-1 antibody after anti-OX40 antibody could produce synergistic antitumor effects in animal models, indicating the importance of the administered sequence of anti-PD-1 antibody in combined cancer immunotherapy (9). Theoretically, the sequence of anti-PD-1 antibodies and chemotherapeutic agents administered may also affect the efficacy. PD-1 blockade may result in the expansion of tumor-specific T cells (10); proliferating cells are more likely to be killed by chemotherapeutic drugs, so it may reduce the possibility of chemotherapy drugs to kill T cells by administered anti-PD-1 antibodies after chemotherapy drugs. Therefore, it is imperative to explore the administered sequence of anti-PD-1 antibodies and chemotherapeutic agents. Thus, we designed this study to explore the sequence of chemotherapy and toripalimab on the pathological complete response (pCR) rate and safety in patients with locally advanced ESCC.

## Patients and Methods

This study was approved by the Ethics Committee of Henan Cancer Hospital and was conducted in accordance with the Declaration of Helsinki and Good Clinical Practice Guidelines. All enrolled patients provided written informed consent.

### Patients

This was a randomized, open-label, single-center, phase II study. The eligibility criteria for patients eligibility were thoracic ESCC confirmed by pathology, without distant metastasis, resectable or potentially resectable as assessed by thoracic surgical experts, ECOG 0-1, clinical stage II/III/IVa, age 18–70 years, adequate organ function, and no history of other malignant tumors. The exclusion criteria were severe infection within 4 weeks before enrollment, bronchial asthma requiring intermittent use of bronchodilators or medical intervention, use of immunosuppressants for coexisting diseases, obvious cardio-cerebrovascular diseases, severe allergic constitution, severe mental disorders, abnormal coagulation function, pulmonary fibrosis, interstitial pneumonia, pneumoconiosis, pregnant or lactating women, and not meeting the criteria for enrollment evaluated by investigators.

### Pretreatment Stage

All patients underwent thoracoabdominal and pelvic enhanced CT, esophageal enhanced MRI, and endoscopic ultrasound for accurate clinical staging. Endoscopic ultrasound was used for the T stage. Enhanced MRI was used for the N stage and for measuring the length of the diseased esophagus in a diffusion-weighted imaging (DWI) sequence (11).

### Neoadjuvant Therapy

Patients were enrolled by investigators and randomly assigned (1:1) according to the enrollment order. Toripalimab (a type of anti-PD-1 antibody produced by Suzhou Zhonghe Biomedical Technology Co., Ltd., Suzhou, China) was administered at a fixed dose of 240 mg every cycle. Paclitaxel was administered at a dose of 150–175 mg/m^2^ every cycle, and cisplatin was administered at a dose of 70–75 mg/m^2^. Neither the investigators nor the patients were masked to treatment allocation. The patients in the experimental group received paclitaxel and cisplatin on day 1 and toripalimab on day 3. The patients in the control group received paclitaxel and cisplatin and toripalimab on day 1. The treatment cycle was 21 days in the control group and 21–28 days in the experimental group. Radical surgery was performed 4–6 weeks after the second chemotherapy plus toripalimab.

### Surgery

McKeown esophagogastrectomy or thoracoscopic McKeown esophagogastrectomy was performed for upper and middle thoracic esophageal cancer, and Ivor Lewis esophagogastrectomy or thoracoscopic Ivor Lewis esophagogastric resection was used for middle and lower esophageal cancer.

### Pathological Evaluation

The depth of tumor invasion, lymph node metastasis, and surgical margin were evaluated according to the American Joint Committee on Cancer Criteria for esophageal carcinoma (12). The extent of the residual tumors was divided into four categories: grade 0, no evidence of viable tumor cells (pCR); grade 1, single cells or rare small groups of cancer cells (near-complete response); grade 2, residual cancer cells with evident tumor regression, but more than single cells or rare small groups of cancer cells (partial response); and grade 3, extensive residual cancer without evident tumor regression (poor or no response) (13). The postoperative pathological evaluation was carried out by two experienced pathologists. The expression of PD-L1 was examined using the Dako 22C3 antibody and counted based on a CPS.

### Objectives and Endpoints

The primary objective of this study was to explore the influence of sequence of toripalimab and chemotherapy on pCR rate in locally advanced ESCC. The secondary objectives were to explore the safety of chemotherapy and toripalimab as neoadjuvant therapy and the treatment-related adverse events (AEs), which were evaluated according to the National Cancer Institute Common Terminology Criteria for Adverse Events Version 5.0 (NCI-CTC AE 5.0) and the influence of PD-L1 expression on pCR.

### Statistics

The sample size was calculated by PASS ver. 15.0. The pCR of toripalimab and chemotherapy administered on the same day was about 7%; it was speculated that toripalimab delayed on day 3 could increase the pCR to 35%. With a one-side α ≤ 0.05 as the test level, the sample size of 30 cases was needed when β = 0.2. All the statistical analyses were conducted using SPSS Statistics ver. 22.0 (IBM Corp., Armonk, NY, USA). The patients were divided into two groups according to their enrollment order. Continuous variables were calculated as means with SD or medians with ranges. Categorical variables were calculated as frequencies. A t-test was performed to compare continuous variables, and a chi-square test was performed for categorical variables. A two-sided *p*-value of <0.05 was considered statistically significant. All statistical analyses were performed using SPSS 22.0 (IBM Corp., Armonk, NY, USA).

## Results

### Patients

**Figure 1:**
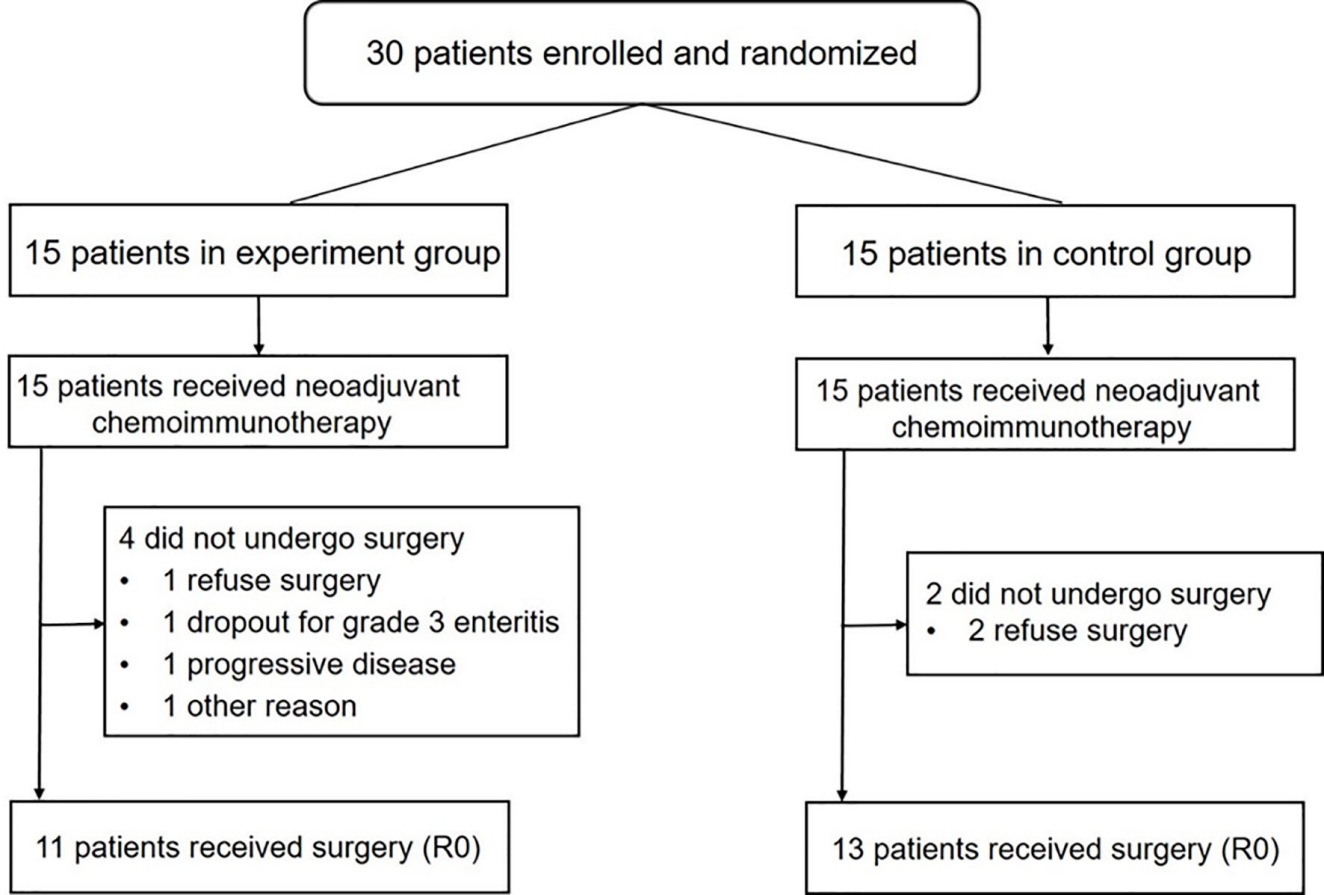
Consolidated Standards of Reporting Trials (CONSORT) diagram.

Thirty patients were enrolled between July 2019 and October 2020. The Consolidated Standards of Reporting Trials (CONSORT) reporting procedures are presented in **Supplementary Table 1**. The baseline characteristics of the patients are listed in **Table 1**. After neoadjuvant therapy, 24 patients underwent surgery. The flowchart of the study is presented in **Figure 1**.

**Table 1:** Baseline characteristics of different groups.

Characteristics	Group		Statistics	*p*-Value
	Experimental group (N = 15)	Control group (N = 15)		
Age (year)	63.80 ± 6.07	63.13 ± 5.05	F = 0.33	0.746
Gender, n (%)			×^2^ = 2.73	0.215
Male	13 (86.67%)	9 (60.00%)		
Female	2 (13.33%)	6 (40.00%)		
T			×^2^ = 2.73	0.215
3	9 (60.00%)	13 (86.67%)		
4	6 (40.00%)	2 (13.33%)		
N			×^2^ = 0.286	0.867
0	4 (26.67%)	3 (20.00%)		
1	8 (53.33%)	8 (53.33%)		
2	3 (20.00%)	4 (26.67%)		
Stage			×^2^ = 1.815	0.404
II	3 (20.00%)	3 (20.00%)		
III	7 (46.67%)	10 (66.67%)		
IVa	5 (33.33%)	2 (13.33%)		
ECOG PS			×^2^ = 1.22	0.462
0	8 (53.33%)	5 (33.33%)		
1	7 (46.67%)	10 (66.66%)		

ECOG PS, Eastern Cooperative Oncology Group performance status.

### Efficacy

Among the 30 patients, two did not undergo radiological evaluation and surgery after two neoadjuvant therapy cycles because of the COVID-19 pandemic, and one patient withdrew from the study because of immune-associated enteritis. The radiological efficacy of neoadjuvant therapy was evaluated in the remaining 27 patients. The Response Evaluation Criteria in Solid Tumors (RECIST) objective response rate (ORR) was 66.7% (18/27 patients). **Supplementary Figure 1** shows the tumor burden changes from baseline.

**Figure 2:**
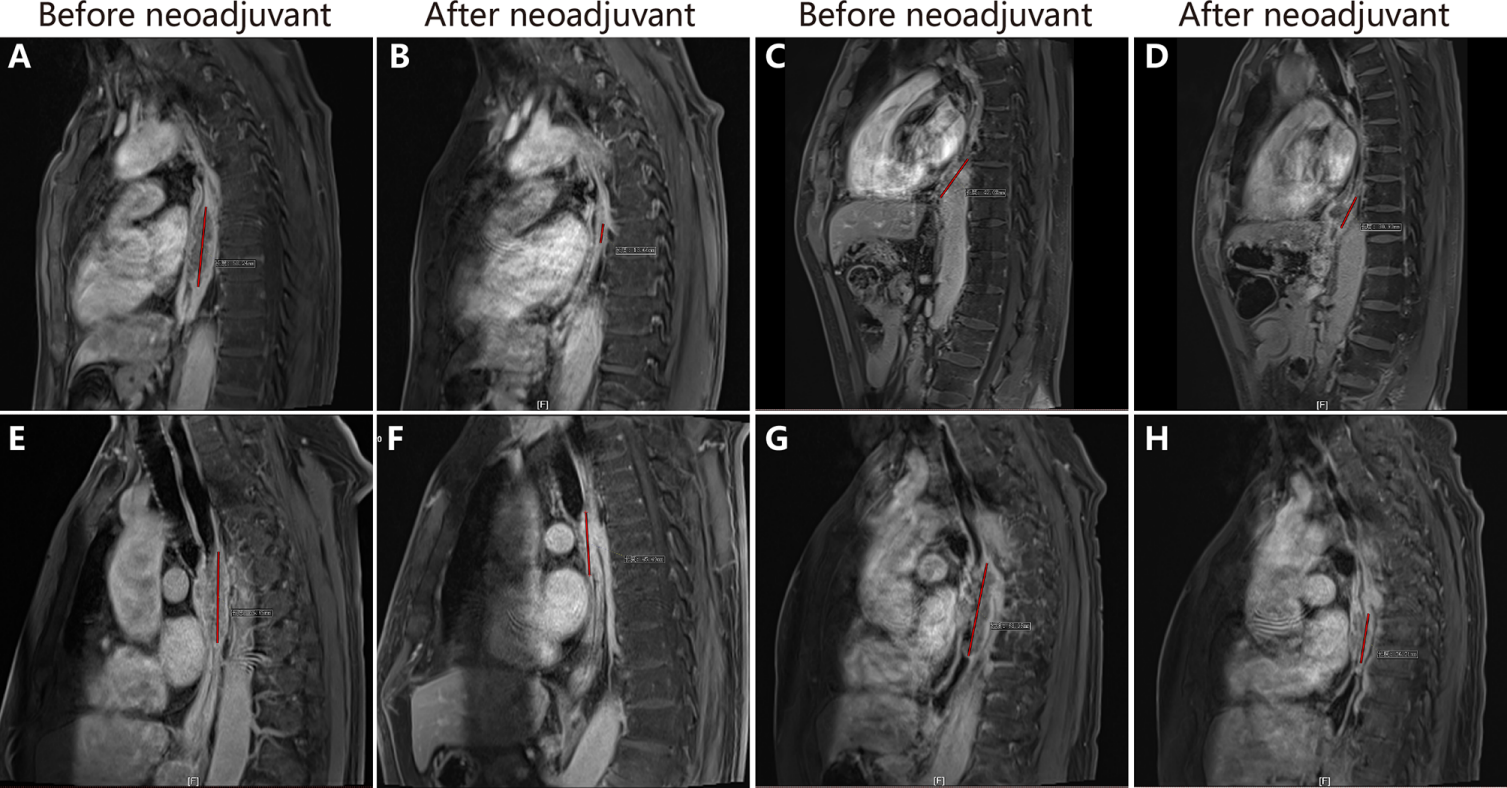
The changes of esophageal lesions in magnetic resonance imaging. **(A, B)** show the esophageal lesion in patient 4 before and after neoadjuvant chemoimmunotherapy, the lesion reduced significantly. **(C, D)** show esophageal lesion in patient 7 before and after neoadjuvant chemoimmunotherapy. **(E, F)** show esophageal lesion in patient 1 before and after neoadjuvant chemoimmunotherapy. **(G, H)** show esophageal lesion in patient 8 before and after neoadjuvant chem.

Eleven patients in the experimental group and 13 patients in the control group underwent radical resection. R0 resection was performed in all these 24 patients. Five patients (20.8%) achieved pCR: four patients in the experimental group and one patient in the control group achieved pCR. The difference in pCR between the two groups was not statistically significant (χ^2^ = 3.092, *p* = 0.079). Neoadjuvant chemotherapy plus toripalimab did not increase the difficulty in surgery, and the median number of lymph nodes excised was 23 (range 15–49). The numbers of resected lymph nodes were 21 and 26 in the experimental group and control group, respectively (F = 0.669, *p* = 0.422). **Figure 2** shows the esophageal lesions on MR of patients with or without pCR before and after neoadjuvant therapy in the two groups.

**Figure 3:**
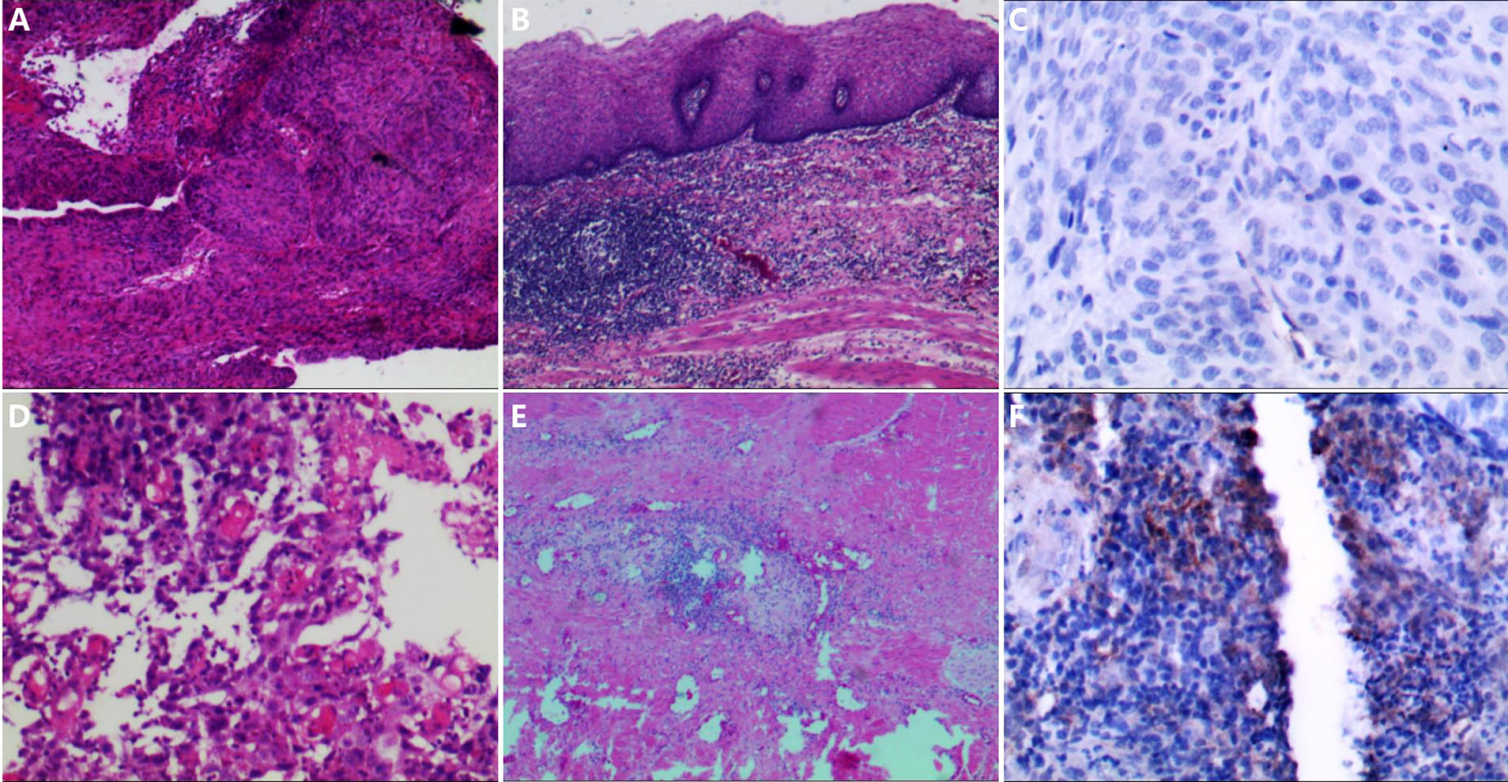
The pathological changes before and after neoadjuvant chemoimmunotherapy and the PD-L1 expression. **(A, B)** The pathological changes in patient 1; after neoadjuvant chemoimmunotherapy, the tumor cells disappeared, and many lymphocytes infiltrated. **(C)** The expression of PD-L1 combined positive score (CPS) in the sample before therapy in patient 1; and the expression of PD-L1 CPS was lower than 1. **(D, E)** The pathological changes in patient 4; after neoadjuvant chemoimmunotherapy, the tumor cells disappeared, and some lymphocytes infiltrated. **(F)** The expression of PD-L1 CPS in the sample before therapy in patient 4, and the expression of PD-L1 CPS was 10.

**Figure 4:**
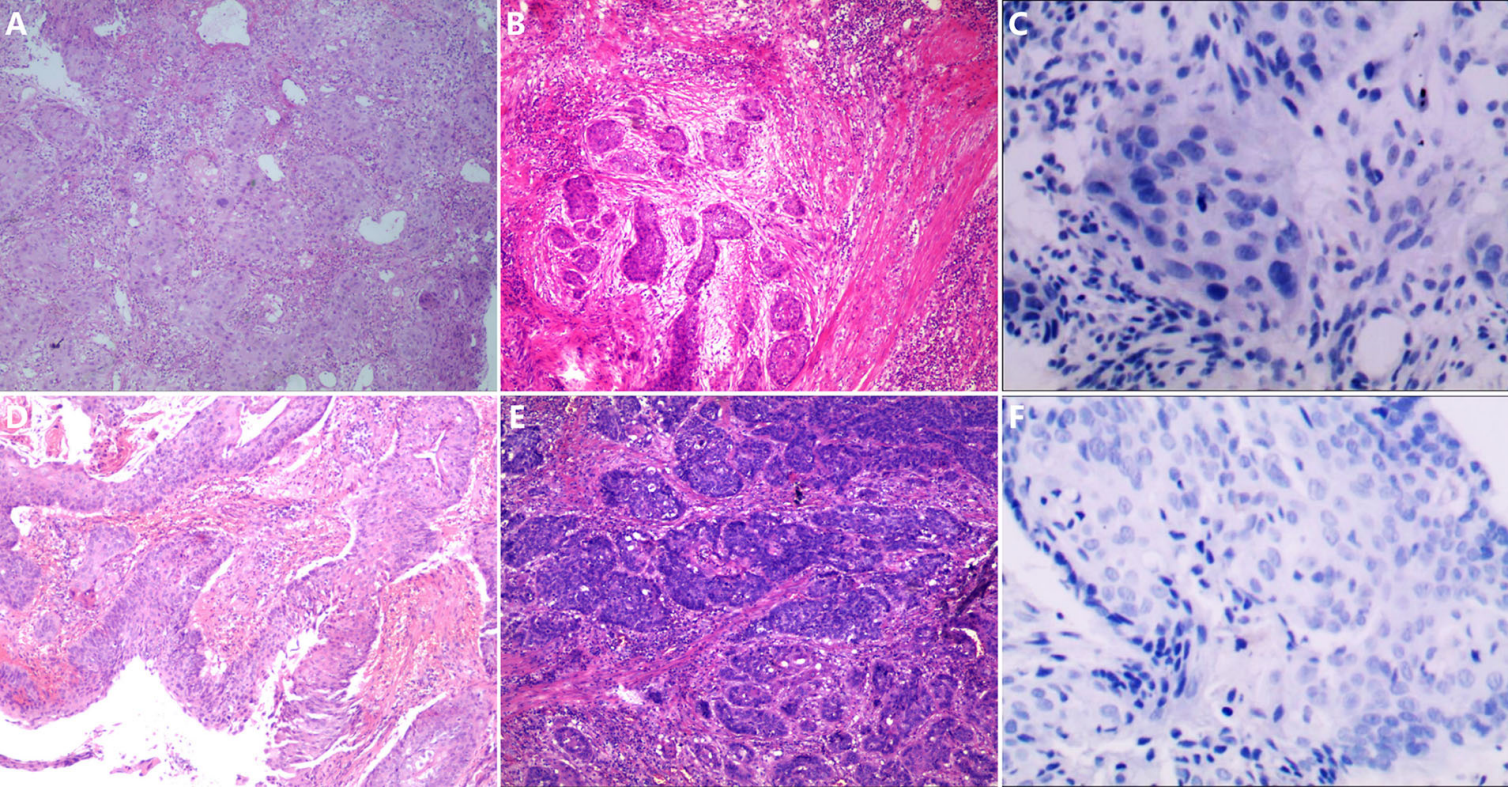
The pathological changes before and after neoadjuvant chemoimmunotherapy and the PD-L1 expression. **(A, B)** The pathological changes in patient 7; after neoadjuvant chemoimmunotherapy, there are a large number of tumor cells left in the section, and a few lymphocytes infiltrated. **(C)** The expression of PD-L1 combined positive score (CPS) in the sample before therapy in patient 7, and the expression of PD-L1 CPS was lower than 1. **(D, E)** The pathological changes in patient 8; after neoadjuvant chemoimmunotherapy, there are a large number of tumor cells left in the section and few lymphocytes infiltrated. **(F)** The expression of PD-L1 CPS in the sample before therapy in patient 8, and the expression of PD-L1 CPS was lower than 1.

PD-L1 CPS examinations were performed with the tissue samples of 14 patients (seven in each group) whose tissue was enough before surgery. Only one patient in the control group had a PD-L1 CPS of 10, while the others had PD-L1 CPS ≤1. The patient with PD-L1 CPS of 10 achieved pCR. Two of the six patients with PD-L1 CPS ≤1 in the experimental group achieved pCR, while none of the six patients with PD-L1 CPS ≤1 in the control group achieved pCR. **Figure 3** shows the expression of PD-L1 CPS with pCR in both groups and according to pathological appearance before therapy and after surgery. **Figure 4** shows the expression of PD-L1 CPS with non-pCR in both groups and according to pathological appearance before therapy and after surgery.

### Safety

During the period of neoadjuvant therapy, almost all the patients developed treatment-related AEs. The most common AEs were nausea, vomiting, and peripheral neurotoxicity, which can be attributed to cisplatin and paclitaxel. Notably, one patient in the experimental group developed grade 3 immune-associated enteritis after one cycle of therapy, and this patient recovered after glucocorticoid and plasma exchange therapy. Postoperative pulmonary infection occurred in 13 patients, during which three developed sepsis and needed application of broad-spectrum antibiotics: two of the patients recovered from sepsis, and one died 20 days after surgery. The patient who died from sepsis developed troponin elevation, which was suspected to be due to immune-related myocarditis during the course of sepsis. Anastomotic fistula occurred in two patients, one in the experimental group and one in the control group, both patients recovered from anastomotic fistula, and neither of them underwent secondary surgery because of anastomotic fistula. The detailed treatment-related AEs and postoperative complications are shown in **Table 2**. There was no difference in treatment-related AEs and postoperative complications between the two groups.

**Table 2:** Treatment-related adverse events and postoperative complications.

Characteristics	Experimental group		Control group		×^2^	*p*-Value
	Grade 1/2	Grade 3/5	Grade 1/2	Grade 3/5		
Nausea and vomiting	12		15		3.330	0.068
Diarrhea	2		3		0.240	0.624
Mouth ulcers	1				1.034	1.034
Fatigue	1				1.034	0.309
Dizzy	1				1.034	0.309
Neuromuscular toxicity	3		5		0.682	0.409
Hair loss	8		9		0.136	0.713
Leukopenia	4		4	2	0.600	0.439
Thrombocytopenia	3		1		1.154	0.283
Elevated transaminase			1		1.034	0.309
Hypoglycemia			1		1.034	0.309
Hypokalemia	2	1	1	3	0.186	0.666
Hyponatremia	1			1	0.000	1.000
Hypocalcemia			1		1.034	0.309
Immune-related enteritis		1			1.034	0.309
Immune-related myocarditis			1		1.034	0.309
Pulmonary infection	4	1	7	1	1.22	0.269
Anastomotic fistula	1		1		0.000	1.000
Pneumothorax			1		0.216	0.642
Subcutaneous emphysema	1		2		0.883	0.347

## Discussion

To the best of our knowledge, in this study, we present for the first time that the sequence of chemotherapy and toripalimab might influence the pCR in patients with locally advanced ESCC, with relatively manageable treatment-related AEs. The pCR was 36% in the experimental group and 7% in the control group, suggesting that the different sequences of administration of chemotherapy and toripalimab might affect the clinical efficacy. The regimen was well-tolerated, and the AEs of neoadjuvant chemoimmunotherapy were similar to those reported in the studies on advanced-stage esophageal cancer (6, 14). No new AEs occurred during the treatment. The most common AEs in the stage of neoadjuvant therapy were mainly caused by chemotherapeutic agents. One patient developed troponin elevation during pulmonary infection after surgery, and this patient died from severe pulmonary infection. One patient withdrew from the study because of immune enteritis in the stage of neoadjuvant therapy. These findings suggested that we should pay much attention to the emergence of immune-related AEs, especially those uncommon AEs during treatment.

Studies conducted in patients with ESCC receiving neoadjuvant concurrent chemoradiotherapy showed that 18 of the 37 patients achieved pCR, and the rate of pCR was 49%, while the occurrence of AEs ≥grade 3 was as high as 25% (3). Patients whose tumors achieved pCR to neoadjuvant therapy had significantly longer relapse-free survival and overall survival (15). Since the factors influencing therapeutic regimens for esophageal cancer between Eastern and Western countries are different, therapeutic strategies adopted in Asian countries, especially in Japan and China, often differ from those in Western countries (16). The JCOG9907 study compared the clinical efficacy of neoadjuvant chemotherapy and adjuvant chemotherapy in patients with stage II/III ESCC, and the results showed that the 5-year survival rate of patients who received neoadjuvant chemotherapy was significantly higher than in those who received adjuvant chemotherapy. There was no increase in postoperative complications and hospital mortality (4, 5). Therefore, at present in Japan, patients with stage II/III ESCC are recommended for neoadjuvant chemotherapy followed by surgery or simultaneous chemoradiotherapy. In China, there is no standard regimen for patients with stage II/III ESCC; the recommended regimen for this kind of patients is to enroll in clinical studies.

In China, a randomized clinical trial in patients with locally advanced ESCC comparing the neoadjuvant chemoradiotherapy with neoadjuvant chemotherapy showed that the pCR rates were 35.7% and 3.8% individually, and there was no significant difference in postoperative morbidity between the two groups (47.4% vs. 42.6%) (17). The pCR rate of neoadjuvant chemotherapy in the Chinese population with locally advanced ESCC is relatively low and needs improvement. If the performance status of patients is good, simultaneous chemoradiotherapy might be a good choice. With the development of immunotherapy, clinical trials based on immunotherapy are also conducted in a neoadjuvant setting in such patients. In PALACE-1 study, pembrolizumab was added in chemoradiotherapy for ESCC, the rate of pCR was 55.6% (10/18), grade III and higher AEs occurred in 13 patients (13/20, 65%), and one patient died (18). Simultaneously, the addition of atezolizumab in neoadjuvant chemoradiotherapy in patients with resectable esophageal adenocarcinoma showed that the rate of pCR was 25%, grade 3/4 AEs was observed in 40% (16/40) patients, and one patient died (19). The incidence of grade III and higher AEs in patients with locally advanced ESCC treated with neoadjuvant concurrent chemoradiotherapy combined with anti-PD-1/PD-L1 antibodies was high. The neoadjuvant therapeutic regimen containing immunotherapy in patients with locally advanced ESCC needs to be optimized. In Chinese patients, neoadjuvant chemoimmunotherapy might be one of the choices (4, 5, 17, 18).

When the anti-PD-1 antibody is combined with other kinds of therapies such as chemotherapy, radiotherapy, anti-angiogenesis, or immunomodulator, the sequence of anti-PD-1 antibody and other regimens might influence the clinical efficacy (7, 20–22). The TONIC trial was a non-comparative phase II study to explore whether induction treatment could induce a more inflamed tumor microenvironment. Sixty-seven patients with metastatic triple-negative breast cancer were randomized to nivolumab without induction or with 2-week low-dose induction with irradiation or chemotherapy; the results showed that the ORRs in the doxorubicin cohort, cisplatin cohort, cyclophosphamide cohort, radiotherapy cohort, and no induction cohort were 35%, 23%, 8%, 8%, and 17%, respectively. Immune-related genes involved in PD-1/PD-L1 and T-cell cytotoxicity pathways were upregulated in the doxorubicin and cisplatin cohorts (23). These results indicated that preconditioning with some chemotherapeutic agents could induce a more inflamed tumor microenvironment, which would favor immunotherapy. GeparNuevo study was a phase II study that investigates whether the addition of durvalumab into neoadjuvant chemotherapy increases the pCR rate in patients with early triple-negative breast cancer: the patients were divided into three cohorts, and the pCR rate with durvalumab was 53.4% versus placebo 44.2% (*p* = 0.287). In the window-phase cohort, the pCR rate was 61% versus 41.4% (*p* = 0.048) (21). This study showed again that preconditioning with durvalumab 2 weeks before chemoimmunotherapy could improve the pCR rate, which showed the importance of the administration sequence of immunotherapy and chemotherapy. Therefore, when designing a chemoimmunotherapy regimen, the doses, sequence, and administration intervals should be considered. Considering the mechanisms of synergy between chemotherapy and immunotherapy, sequential approaches might be helpful (7), and there was a retrospective study that showed that administering PD-1/PD-L1 inhibitors 1–10 days (especially 3–5 days) after chemotherapy is superior to administering PD-1/PD-L1 inhibitors before or concurrent with chemotherapy in patients with refractory lung cancer (22).

The mechanisms of postponing the application of anti-PD-1 antibody for 2 days when combined with chemotherapy with anti-PD-1 antibody may be as follows: studies in preclinical animal models found that cyclophosphamide could upregulate PD-1 and PD-L1 expression on tumor-specific CD8+ T cells in tumor-bearing C3 mice. Then the application of anti-PD-1 treatment could increase the antitumor effect, thus increasing the systemic antitumor effect (24). Short-term chemotherapy in patients with triple-negative breast cancer may induce a more favorable tumor microenvironment and increase the likelihood of response to PD-1 blockade therapy (21). In lung squamous cell cancer and colon cancer, there is a synergistic effect induced by low-dose chemotherapy followed by anti-PD-1 antibodies (25, 26). Therefore, theoretically, the effect of using PD-1 antibodies after chemotherapy may be better than that when using them simultaneously, but this does not imply that the later use of an anti-PD-1 antibody might yield better efficacy. Studies in patients with brain metastasis of melanoma have found that the immunotherapeutic efficacy within 4 weeks after stereotactic radiosurgery treatment is better than that after 4 weeks (20). The subgroup analysis of the PERCIFIC study also showed that the efficacy of anti-PD-L1 was better in the group in which anti-PD-L1 was applied within 2 weeks after the end of simultaneous chemoradiotherapy (27). A clinical study showed that earlier application of anti-PD-1 antibody is better (10). Therefore, we postponed the application of anti-PD-1 antibody for 2 days after chemotherapy. When the anti-PD-1 antibody was applied alone, the T cells with antitumor activity in peripheral blood reached the peak at 1 or 2 weeks after the first dose of anti-PD-1 antibody and then decreased gradually (28). To reduce the influence of the second cycle of chemotherapy on activated T cells, the immune-chemotherapy cycle in the experimental group was 3–4 weeks. In this study, a trend of higher pCR rate was observed in the experimental cohort than that in the control cohort (χ^2^ = 3.092, *p* = 0.079), indicating the importance of the sequence of chemotherapy and immunotherapy.

Thus far, there were no biomarkers for predicting the efficacy of anti-PD-1 therapy in esophageal cancer, although PD-L1 expression has been used to guide anti-PD-1 therapy in non-small cell lung cancer without driver gene mutations (29). In KEYNOTE 590 study, patients with PD-L1 CPS > 10 could benefit more from chemotherapy combined with pembrolizumab, while the risk of death in patients with CPS < 10 could also be reduced (6). Patients could benefit from anti-PD-1 therapy regardless of the expression of PD-L1 in ATTRACTION-3 and ESCORT studies (14, 30). In this study, we examined the expression of PD-L1 CPS in 14 patients (seven in each group) with the samples before treatment; the results showed that almost all the patients had PD-L1 CPS ≤1 (except one with PD-L1 CPS 10), which indicated that our patients might represent the group of patients insensitive to anti-PD-1 therapy. Two of the six patients with PD-L1 CPS ≤1 in the experimental cohort achieved pCR, and the rate of pCR in the experimental group was 36.4%; it might be even higher if we could avoid the influence of COVID-19. A higher rate of pCR might be obtained by delaying anti-PD-1 antibody application by 2 days when chemotherapy plus anti-PD-1 therapy was used.

There are several limitations of this study. The first was the influence of COVID-19 from February to May in 2020. The chemoimmunotherapy cycles were forcibly prolonged, and the operations were postponed in several patients, which may be one of the reasons that the rate of pCR in this study was lower than that reported previously (18, 31–33). Second, as this study was a small-sample exploratory study, we initially planned to enroll 30 patients. Because the representation of the small sample was not strong, bias was inevitable in the process of selecting patients. The PD-L1 expression results showed that only one patient with PD-L1 CPS was 10, and the others were PD-L1 ≤ 1, which suggested that the patients in this study may only present the group of patients insensitive to anti-PD-1 therapy. Third, due to the limitation of the preoperative specimens, not all the patients have enough tissue specimens for PD-L1 CPS examination. Last, there was no exploratory study on other biomarkers that might affect the efficacy. However, our results showed that the pCR rate in patients with delayed application of anti-PD-1 antibody was 36.4%. There was a statistical boundary difference, suggesting that when chemotherapy combined with anti-PD-1 antibody was used in a neoadjuvant setting, delaying anti-PD-1 antibody therapy by 2 days might have greater clinical benefits.

In summary, our results showed that neoadjuvant chemotherapy plus toripalimab is safe and effective in patients with locally advanced ESCC, with a pCR rate of 20.8%. In the process of neoadjuvant chemotherapy combined with anti-PD-1 antibody, the application of anti-PD-1 antibody 2 days after chemotherapy may be more conducive to the synergistic effects. Further clinical studies are needed in a larger sample of patients to verify the influence of different administration sequences on pCR and explore its underlying biomarkers. We are conducting a phase III study to compare the clinical efficacy of neoadjuvant chemoimmunotherapy with that of neoadjuvant chemotherapy for resectable esophageal cancer (NCT 04280822).

## Data Availability Statement

The raw data supporting the conclusions of this article will be made available by the authors, without undue reservation.

## Ethics Statement

The studies involving human participants were reviewed and approved by Henan Cancer Hospital Ethics Committee. The patients/participants provided their written informed consent to participate in this study.

## Author Contributions

QG and JZ took part in the conceptualization. WX, LZ, YaZ, and LL carried out writing—original draft preparation. QG, XR, and ZW carried out writing—review and editing. TL, HL, and BM carried out the statistical analyses and visualization. XC and HZ performed the pathological evaluation and PD-L1 examination. JQ and YoZ performed the imaging evaluation. BL, XL, GL, DY, YY, YS, and XF performed the data collection. All authors contributed to the article and approved the submitted version.

## Funding

This study was supported by the National Natural Science Foundation of China (grant numbers 81902902 and 81972802).

## Conflict of Interest

The authors declare that the research was conducted in the absence of any commercial or financial relationships that could be construed as a potential conflict of interest.

## Publisher’s Note

All claims expressed in this article are solely those of the authors and do not necessarily represent those of their affiliated organizations, or those of the publisher, the editors and the reviewers. Any product that may be evaluated in this article, or claim that may be made by its manufacturer, is not guaranteed or endorsed by the publisher.

## Abbreviations

pCR, pathological complete response; ESCC, esophageal squamous cell carcinoma; CPS, combined positive score; AE, adverse events; NCI-CTC, National Cancer Institute Common Terminology Criteria; ORR, objective response rate.
